# Transcranial Focused Ultrasound (tFUS) and Transcranial Unfocused Ultrasound (tUS) Neuromodulation: From Theoretical Principles to Stimulation Practices

**DOI:** 10.3389/fneur.2019.00549

**Published:** 2019-06-11

**Authors:** Lazzaro di Biase, Emma Falato, Vincenzo Di Lazzaro

**Affiliations:** ^1^Neurology, Neurophysiology, and Neurobiology Unit, Department of Medicine, Campus Bio-Medico University of Rome, Rome, Italy; ^2^Unit of Neurophysiology and Neuroengineering of Human-Technology Interaction, School of Medicine, Campus Bio-Medico University of Rome, Rome, Italy

**Keywords:** focused ultrasound, transcranial stimulation, non-invasive brain stimulation (NIBS), transcranial focused ultrasound (tFUS), transcranial ultrasound (tUS)

## Abstract

Transcranial focused ultrasound is an emerging technique for non-invasive neurostimulation. Compared to magnetic or electric non-invasive brain stimulation, this technique has a higher spatial resolution and can reach deep structures. In addition, both animal and human studies suggest that, potentially, different sites of the central and peripheral nervous system can be targeted by this technique. Depending on stimulation parameters, transcranial focused ultrasound is able to determine a wide spectrum of effects, ranging from suppression or facilitation of neural activity to tissue ablation. The aim is to review the state of the art of the human transcranial focused ultrasound neuromodulation literature, including the theoretical principles which underlie the explanation of the bioeffects on neural tissues, and showing the stimulation techniques and parameters used and their outcomes in terms of clinical, neurophysiological or neuroimaging results and safety.

## Introduction

Preliminary animal studies suggest that, potentially, different sites in the peripheral nervous system, from nerves (1) to spinal roots (2), and in the central nervous system, from superficial regions like primary motor cortex (3) or frontal eye field (4), to more deep areas like hippocampus (3), amygdala (5), or thalamus (6) can be targeted by focused ultrasound stimulation technique. In addition, animal studies showed that this technique has a high spatial resolution, useful also for mapping small brain areas, as shown by Fry (7) for the mapping of lateral geniculate nucleus, or by Ballantine et al. (2) for the stimulation of Edinger-Westphal nucleus.

Furthermore, a recent fMRI resting-state functional connectivity animal study (8), showed that the effect of tFUS neuromodulation can last for up to 2 h after stimulation, opening a new way to explore not only the online effect but also the long lasting effect of neuromodulation. The first human transcranial application of ultrasounds for neuromodulation was described by Hameroff et al. (9), with an unfocused transcranial ultrasound (tUS) continuous stimulation of posterior frontal cortex, applied on 31 patients affected by chronic pain. The first human application of focused transcranial ultrasound (tFUS) technique was described by Legon et al. (10). They targeted the primary somatosensory cortex of healthy volunteers, in a within-subjects, sham-controlled study. One of the most interesting results of tFUS applications was a case report of emergence from minimally conscious state, after low intensity non-invasive ultrasonic thalamic stimulation in a patient after acute brain injury (11). Following this first single evidence, a clinical trial is ongoing to explore the effect of thalamic low intensity focused ultrasound in acute brain injury patients (12).

Regarding peripheral nervous system neuromodulation, Bailey et al. (13) explored the ability of continuous US at 1.5 MHz in modulating the ulnar nerve stimulation response to magnetic stimulation (MS). This study showed no significant change in electromyographic response during magnetic plus US ulnar nerve stimulation. However, further studies are needed in order to explore different parameter of stimulation.

In recent years, the scientific community showed a progressive increasing interest on FUS neuromodulation, and some reviews have been published in order to summarize the state of the art on this topic (14–18).

### Mechanisms of Actions of US Neuromodulation

Focused ultrasound is a non-invasive, non-ionizing technique. In order to target a brain region, the first challenge is to let ultrasounds single waves to reach the target at the same time, without different acoustic reflection, refraction, and distortion due to the inhomogeneity of skull bone. This problem can be solved by time shifting each single ultrasound wave, according to the related skull bone acoustical properties, in order to let all the waves to reach the target at the same time (19–22).

The mechanical interaction between US and neuronal membranes can modify the membrane gating kinetics through the action on mechanosensitive voltage-gated ion channels or neurotransmitter receptors (23–25). The study of Tyler et al. (25) supports this hypothesis. Their study showed, on *ex vivo* mouse brains and hippocampal slice cultures, that low-intensity, low-frequency ultrasound (LILFU) is able to activate voltage-gated sodium and calcium channels. However, this can't be the only mechanism of action, explaining the action potential induction, since in simulations, considering the role of membrane tension on activation of mechanically sensitive voltage gated channels, the resulting effect was too low to induce an excitation (26, 27).

In addition, the mechanical action of US is able to induce cavitation into the cellular membrane, by means of membrane pore formation, which changes the membrane permeability.

**Figure 1:**
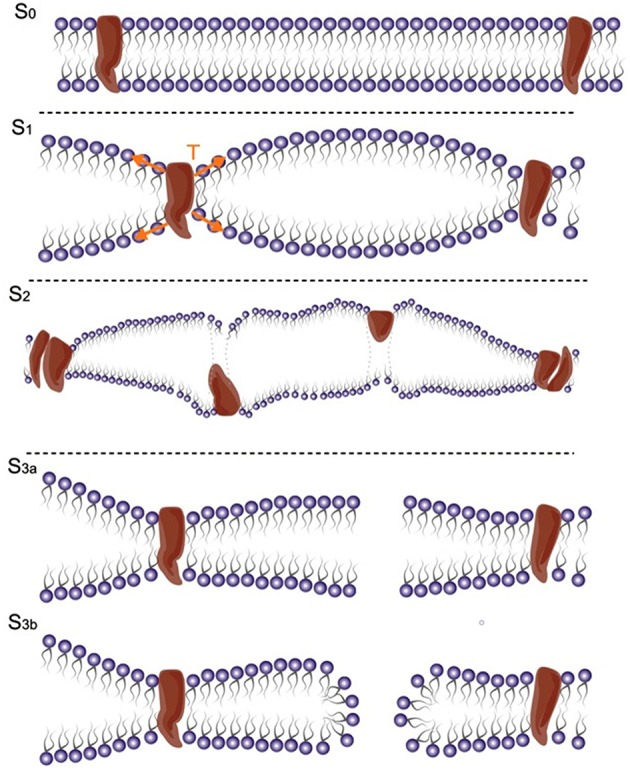
Ultrasound gradually increases tension in the membrane. From the reference stage (S0), the stretch first activates mechanosensitive proteins (S1); growing tension might damage membrane proteins (S2) and then might induce pore formation (S3a, S3b) or cause membrane rupture [modified, with permission, from Krasovitski et al. (28)].

The bilayer sonophore model (28) was introduced to better explain the bioeffects of US, taking into consideration the biomechanical proprieties of US and of cell membranes. According to this model (28), the mechanical energy of US leads to periodic expansions and contractions of the membrane. In this model, the US bioeffect is dependent on the tension applied to the membrane. With a progressive increase in membrane stretch intensity, the bioeffect is mediated by different mechanisms. First by the activation of mechanosensitive proteins. Then, with an increase of intensity, there is a pore formation and with the maximum stretch that can be achieved with the technique a membrane rupture and irreversible lesion is obtained (28) (Figure 1).

**Figure 2:**
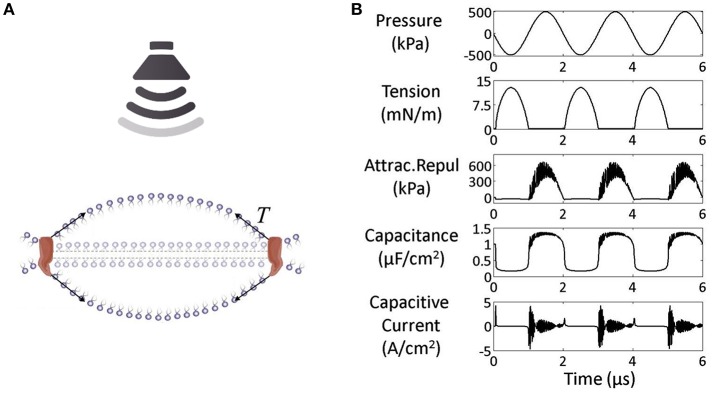
**(A)** Under US effect the membrane starts fluctuating around a steady state. **(B)** Mechano-electrical dynamics of the model membrane to US (pressure amplitude 500 kPa and frequency 0.5 MHz): The increase in Acoustic pressure induces an increase in attraction/repulsion force, which increases the capacitance leading finally to a capacitive current. Acoustic pressure (kPa), tension (mN/m), combined attraction/repulsion force per area between the leaflets (sum of molecular and electrostatic forces, kPa), membrane capacitance (μF/cm^2^), and capacitive displacement current (A/cm^2^) [modified, under the terms of the Creative Commons Attribution 3.0 License, from Plaksin et al. (27)].

Considering the electrical properties of the cell membrane at rest, which can be approximated with a parallel plate capacitor, a hypothesis is that the dynamic fluctuation of the membrane bilayer changes the instantaneous membrane capacitance and leads to a capacitive current, which can potentially activate voltage-dependent sodium and potassium channels (27) (Figure 2). The neuronal bilayer sonophore model (27) combines, in a complementary way, all the biomechanical and bioelectrical proprieties of the cell membrane described, and predicts the stimulation parameter needed to reach a successful motor cortex stimulation. It explains, for example, the higher efficacy of long US stimulation pulses (3, 29, 30), and how the action potential can be elicited after the end of the US stimulus (27, 31), with a good overlap with the experimental results obtained using real stimulation on the mouse motor cortex (30).

### Stimulation Parameters

**Figure 3:**
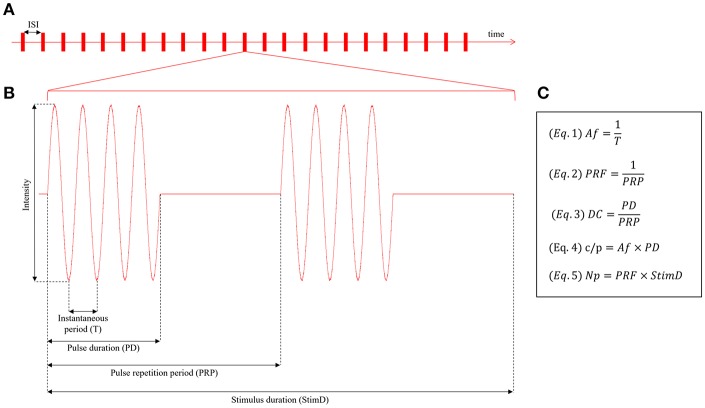
**(A)** Intermittent protocol stimulation. The single sonications are followed by pauses, defined inter stimulation interval (ISI). **(B)** Pulsed paradigm of stimulation, defined by the following parameters: Intensity of stimulation, instantaneous period (T), pulse duration (PD), pulse repetition period (PRP), stimulus duration (StimD). **(C)** Fundamental equations for the stimulation protocol description: Equation (1) = Acoustic frequency (Af), Equation (2) = pulse repetition frequency (PRF), Equation (3) = duty cycle (DC), Equation (4) = cycles per pulse (c/p), Equation (5) = number of pulses (Np).

An acoustic wave can be defined by two fundamental parameters: the intensity, defined as the amplitude of the wave, and the instantaneous period (T), defined as the time needed to complete one single oscillation cycle, which is used to calculate the Acoustic frequency (Af) (Figure 3, Equation 1). In addition to these two parameters, the stimulus duration (StimD) is the total duration of one single sonication.

During the stimulus duration two paradigms of sonication are used: continuous or pulsed. Some of these protocols resemble those used for non-invasive brain stimulation based on repetitive transcranial magnetic stimulation [see Di Lazzaro and Rothwell (32) for a review]. The most used one for neuromodulation is the pulsed paradigm.

For the pulsed paradigm, two additional periods need to be defined: the pulse duration (PD), which is the period of acoustic sonication from the starting point of oscillation to the ending point, before the pause and the pulse repetition period (PRP), which is the period between the starting point of two consecutive sonications, or, in other terms, the sum of the pulse duration (PD) and the pause between two consecutive sonications. This period is used to calculate the pulse repetition frequency (PRF) (Figure 3, Equation 2). For the pulsed paradigm, the duty cycle (DC) (Figure 3, Equation 3) is the fraction of the pulsed repetition period (PRP) covered by the pulse duration (PD). The cycles per pulse (c/p) are the number of cycles during a single pulse (Figure 3, Equation 4); instead, the number of pulses (Np) is the number of pulses throughout the stimulus duration (Figure 3, Equation 5).

The sonication delivered during the stimulus duration period can be repeated, without pauses, for the continuous stimulation protocol. Instead, intermittent protocols are characterized by pauses between the sonications, defined as inter stimulation intervals (ISIs). The intermittent protocol is the most used for FUS neurostimulation, instead the continuous one is the most used for the unfocused stimulation (Table 1).

**Table 1:** tFUS and tUS neuromodulation studies.

**References**	**Device**	**N. of subjects**	**Disease type/healthy subjects**	**Study design**	**Stimulation target**	**Protocol duration**	**Ultrasound parameters**	**Energy**	**Results**	**Adverse events**
Ai et al. (33)	Custom-made, single-element FUS transducer; A_f_: 0.50 MHz Diameter 30 mm, focal length 30 mm, 7T MRI compatible Focused, Pulsed	5	Healthy volunteers	Within-subjects, sham-controlled study	**Primary motor cortex** (tFUS paired with high field 7T fMRI targeted on the dominant thumb BOLD representation)	54 stimuli, ISI 5.5 s	A_f_: 0.50 MHz; PD: 0.36 ms; PRF: 1 kHz; Np: 500; DC: 36%; c/p: 180; StimD: 500 ms	I_SPPA_: 16.95 W/cm^2^; MI: 0.97	tFUS increased BOLD activation volumes generated during a cued tapping task. The effect was spatially confined to the sonicated area. No detectable effects on SMA and PMd.	No auditory or tactile sensation
Legon et al. (34)	Custom- designed, single-element FUS transducer; A_f_: 0.50 MHz Height 1.25 cm, aperture 30 mm, focal length 22 mm, Attached at the center of a TMS 8-coil (Magstim Inc., UK) for concurrent and concentric tFUS/TMS delivery Focused, Pulsed	12 (exp. 1) 10 (exp. 2) 28 (exp. 3)	Healthy volunteers	Within-subjects, sham-controlled study	**Primary motor cortex** (Exp 1–2: dominant FDI hotspot; Exp 3: dominant APB hotspot)	Exp1: 10 tFUS/TMS stimuli from RMT-20% to 100% stimulator output, in increments of 5%, ISI of 10 seconds) Exp2: 10 tFUS/TMS stimulations every 10 s for each TMS paired-pulse ISI from 1 to 15 ms. Exp3: 100 stimuli at random time intervals between 3 and 6 s	A_f_: 0.50 MHz; PD: 0.36 ms; PRF: 1 kHz; Np: 500; DC: 36%; c/p: 180; StimD: 500 ms tFUS 100 ms prior to: the TMS pulse (exp. 1), to the CS (exp. 2) and to the visual stimulus (exp. 3)	I_SPPA_: 17.12 W/cm^2^; I_SPTA_: 6.16 W/cm^2^; MI: 0.9	Concentric and concurrent tFUS/TMS on M1 inhibited the amplitude of single-pulse MEPs, attenuated intracortical facilitation, did not affect intracortical inhibition and significantly reduced reaction time in a motor task.	Mild and moderate symptoms such as neck pain, sleepiness, muscle twitches, itchiness and headache (assessed by questionnaire). No severe symptoms reported.
Legon et al. (35)	Custom-designed, single-element FUS transducer (*Ultran Group, Inc., State College, PA*); A_f_: 0.50 MHz Aperture 63 mm, focal length 70.92 mm (55 mm from exit plane), f# 1.13 Focused, Pulsed	20 (exp. 1) 20 (exp. 2)	Healthy volunteers	Within-subjects, sham-controlled study	**Unilateral sensory thalamus** targeted through a neuronavigation system from the individual MRI	Exp1: 300 stimuli, ISI 4 s Exp2: 90 stimuli	A_f_: 0.50 MHz; PD: 0.36 ms; PRF: 1 kHz; Np: 500; DC: 36%; c/p: 180; StimD: 500 ms Median nerve stimuli time-locked to occur 100 ms after the onset of tFUS waveforms	I_SPPA_: 14.56 W/cm^2^; MI: 0.89 After bone transmission: I_SPPA_: 7.03; W/cm^2^; MI: 0.56	tFUS decreased P14 SEP amplitude. Decrease in ability in a tactile judgement task. Effect upon cortical oscillatory dynamics	Not available
Leo et al. (36)	2 transducers: 1) *3T experiment:* A_f_: 0.50 MHz Active diameter 60 mm, focal length 55 mm, focal FWHM intensity volume 48.64 mm^3^ 2) 7T experiment: A_f_: 0.86 MHz Active diameter 64 mm, focal length 54 mm, focal FWHM intensity volume 35.77 mm^3^ Both: Focused, Pulsed	6 (3T exp.) 1 (7T exp.)	Healthy volunteers	Pre-post interventional study	3T experiment: **Primary motor cortex** hand knob of the dominant hemisphere 7T experiment: **Left head of the caudate**	3T experiment: 90 stimuli, ISI 12-14 s 7T experiment: 5 off/on cycles, stimulation delivered at ISI ≅ 12 s during on cycles	3T experiment: A_f_: 0.50 MHz; PRF: 1 kHz; Np: 500; DC: 36%; c/p: 180; StimD: 500 ms 7T experiment: A_f_: 0.86 MHz; PRF: 1 kHz; DC: 50%; c/p: 420; StimD: 500 ms	I_SPPA_: 6W/cm^2^ (after bone transmission)	tFUS induced BOLD fMRI signals in the targeted cortical regions (in 3 of 6 subjects) and in the targeted subcortical region	Not available
Lee et al. (37)	MRI-compatible FUS transducer Af: 0.27 MHz Focal length 3 cm, acoustic focus 3 mm (diameter) and 17 mm (length) Focused, Pulsed	19 (exp. 1) 10 (exp. 2)	Healthy volunteers	Within-subjects, single-blind, sham-controlled study	**Primary visual cortex**, under 3T MRI guidance	Exp.1: 50 stimuli, ISI 13 s Exp.2: 50 stimuli, ISI 2.5 s	Af: 0.27 MHz; PRF: 500 Hz; PD: 1 ms; DC: 50%; StimD: 300 ms	I_SPPA_: 16.6 W/cm2 Estimates at the target location: I_SPPA_: mean 3 W/cm2; MI: mean 0.6	tFUS induced BOLD fMRI signals in V1 and other visual areas, elicited phosphenes and elicited cortical evoked EEG potentials similar to the classical VEP generated by photic stimulation	No adverse effects, as assessed by neurological examination, anatomical MRI (at 3 time points) and follow-up telephone interviews (after 2 months)
Lee et al. (37)	Two sets of single-element FUS transducers (*Ultran Group Ltd, State College, PA*) Af: 0.21 MHz Shape: segmented-spheres Outer diameter (OD):30 mm Focal distance: 25 mm. Each transducer was affixed to an applicator (*Zamerican, Zacuto, Chicago, IL*) mounted on a helmet (*modified from Giro Section Helmet, Santa Cruz, CA*) Focused, Pulsed	10	Healthy volunteers	Within-subjects, double blind,sham-controlled study	**Left primary and secondary somatosensory cortex** (areas of the hand, separately or simultaneously stimulated under multi-modal neuroimage-guidance)	20 stimuli for each session (4 sessions)	Af: 0.21 MHz; PRF: 500 Hz; PD: 1 ms; DC: 50%; StimD: 500 ms	I_SPPA_: 35.0 W/cm^2^; I_SPTA_: 17.5 W/cm^2^ Estimates at the target location: I_SPPA_: 7.0–8.8 W/cm^2^ I_SPTA_: 3.5–4.4 W/cm^2^	tFUS of either primary and secondary somatosensory cortex, stimulated separately or simultaneously, eliciited tactile sensations from the contralateral hand/arm areas	No abnormal findings post-tFUS (assessed by neurological examination, MMSE, anatomical MRI on the same day, at 2 weeks and 4 weeks, and by telephone interview at 2 months after the sonications)
Monti et al. (11)	BXPulsar 1001, Brainsonix Inc. Single-element spherical transducer; A_f_: 0.65 MHz Diameter and radius of curvature 71.5 mm Focused, Pulsed	1	Post-traumatic disorder of consciousness (minimally conscious state) 19 days post-injury	Case report, part of an ongoing clinical trial (12)	**Thalamus** (MRI-guided by a 3 Tesla Magnetom Tim Trio MR scanner)	10 sonications, 30 s each, separated by 30 s pause intervals	A_f_: 0.65 MHz; PD: 0.5 ms; PRF: 100 Hz	I_SPTA_: 720 mW/cm^2^	Emergence from minimally conscious state	Clinical improvement suggested that the procedure was safe and well-tolerated
Lee et al. (38)	Ceramic piezoelectric FUS transducer (*Channel Industries, Santa Barbara, CA*) Outer diameter 6 cm, radius-of- curvature 7 cm A_f_: 0.25 MHz Low Intensity Focused Ultrasound Pulsation	12 (exp. 1) 6 (exp. 2)	Healthy volunteers	Within-subjects, sham-controlled study	**Primary somatosensory cortex** (hand area) under subject- specific image-guidance	(Exp. 1): 200 stimuli, ISI 3 s (Exp. 2): 100 stimuli, ISI ≅2 s	A_f_: 0.25 MHz; PRF: 500 Hz; Tone-burst-duration: 1 ms; DC: 50%; StimD: 300 ms	I_SPPA_: 3W/cm^2^ Estimated I_SPPA_ at the target: 0.7 ± 0.5 W/cm^2^	tFUS elicited transient tactile sensations on the hand and arm area contralateral to the sonicated hemisphere, with anatomical specificity of up to a finger. EEG showed sonication-specific evoked potentials.	No adverse effects, as assessed by neurological examination, anatomical MRI (at 3 time points) and follow-up telephone interviews (after 2 months)
Mueller et al. (39)	Two-channel, 2 MHz function generator (*BK Precision Instruments*) delivered at 0.5 MHz Focused, pulsed	18 (exp. 1) 7 (exp. 2)	Healthy volunteers	Within-subjects, sham-controlled study	Exp.1 **Somatosensory cortex** (CP3) Exp.2 1cm laterally	120 stimuli, ISI 6 s	A_f_: 0.50 MHz; PD: 0.36 ms; PRF: 1 kHz; Np: 500; c/p: 180; StimD: 500 ms	I_SPPA_: 23.87 W/cm^2^; MI: 1.13	tFUS altered EEG beta phase and modulated the phase rate across beta and gamma frequencies. tFUS affected phase distributions in the beta band of early SEP components. Neuromodulatory effects were lost when the transducer was displaced 1 cm laterally from the original cortical target.	Not available
Legon et al. (10)	Custom-made, single-element FUS transducer; A_f_: 0.50 MHz Diameter 30 mm, focal length 30 mm Focused, Pulsed	10 (exp. 1) 8 (exp. 2) 12 (exp. 3) 12 (exp. 4)	Healthy volunteers	Within-subjects, sham-controlled study	**Primary somatosensory cortex** (crown of the postcentral gyrus and posterior wall of the central sulcus, encephalographic electrode CP3)	Exp 1 and 2: 120 stimuli, ISI 6 s Exp 3: 90 stimuli 100 ms before each task Exp4: 120, ISI 6 s	A_f_: 0.50 MHz; PD: 0.36 ms; PRF: 1 kHz; Np: 500; DC: 36%; c/p: 180; StimD: 500 ms Median nerve stimuli time-locked to occur 100 ms after the onset of tFUS waveforms	I_SPPA_: 23.87 W/cm^2^ (≅4-fold lower through the skull); MI: 1.13 Peak rarefactional pressure: 0.80 MPa	Exp1. A: tFUS significantly attenuated the amplitudes of somatosensory evoked potentials B: tFUS significantly modulated the spectral content of sensory-evoked brain oscillations Exp2. tFUS modulation of brain activity is spatially restricted (≅1 cm or less) Exp3 and 4. tFUS significantly enhanced performance on sensory discrimination tasks without affecting task attention or response bias.	No thermal or mechanical sensation
Gibson et al. (40)	tUS: Phillips CX50 Diagnostic System, with a Phillips S5-1 broadband plane sector transducer array; aperture 20.3cm, frequency range 1–5 MHz. TMS: neuronavigation-assisted eXemia TMS system (*Nextstim Ltd., Helsinki, Finland*) with a 70 mm 8-coil. Unfocused, Continuous	21 (active stim) 22 (sham stim)	Healthy volunteers	Between-subjects, single-blind, sham-controlled study	**Primary motor cortex** (abductor pollicis brevis motor hotspot)	2 min	Af: 2.32 MHz; HGen, B-mode; Harmonics: on; DC: < 1%; Focal depth: 10 cm	Isppa: 34.96 W/cm^2^; Ispta: 132.85 mW/cm^2^; MI: 0.67 Peak negative pressure: 1.02 MPa (in degassed water)	tUS increased cortical excitability (average increase in MEPs amplitude of 33.7% at 1 min and of 32.2% at 6 min post stimulation. No significant differences at 11 and 16 min post stimulation). No differences in mood (assessed by a brief questionnaire on subject well-being)	No significant differences in sensations linked tingling, itching etc. (assessed by questionnaires) between active and sham group
Hameroff et al. (9)	General Electric LOGIQe, 12L-RS probe A_f_: 8 MHz Unfocused, Continuous	31	Chronic pain	Double blind, sham-controlled, crossover study	**Posterior frontal cortex**, contralateral to the maximal pain	15 s stimulation	A_f_: 8 MHz; B Mode; Power: 100%; Depth: 3.5 cm; Harmonics: on; Cross- Xbeam: on	MI = 0.7 Max Intensity = 152 mW/cm^2^ TIs = 0.5 TIc = 0.2 (values at the posterior frontal scalp)	tUS significantly improved measures of global affect derived from subjective reports, at 10 and 40 min following stimulation.	Transient headache exacerbation following stimulation (1 subj)

*In gray background: unfocused stimulation protocols, in white background: focused stimulation protocols. Af, acoustic frequency; c/p, cycles per pulse; DC, duty cycle; Exp, experiment; ISI, inter stimulus interval; ISPPA, Intensity Spatial Peak Pulse Average; ISPTA, Intensity spatial peak temporal average; MEPs, motor evoked potentials; MI, mechanical index; Na, not available; Np, number of pulses; PD, pulse duration (width); PMd, dorsal premotor cortex; PRF, pulse repetition frequency; SI, primary somatosensory cortex; SII, secondary somatosensory cortex; SD, Sonication Duration; SMA, supplementary motor area; StimD, stimulus duration; TI, Thermal Index; Tib, Thermal Index of bone; Tic, Thermal Index of Skull/Cranium; Tis, Thermal Index of soft tissue; TMS, transcranial magnetic stimulation. Note: where not specified, I_SPPA_ is the incident acoustic intensity estimated before transcutaneous and transcranial transmission, e.g., In free water*.

For safety reasons the indexes that describe the thermal and biomechanical effects of the sonication need to be defined. These parameters are related to the instantaneous intensity of stimulation and its instantaneous acoustic pressure. The two main mechanisms that can induce tissue damage are: local heating, which through proteins denaturation leads to cell death, and inertial cavitation. The latter is thought to be mediated by the collapse of gas bubbles due to the pressure exerted by ultrasonic field sufficiently strong to allow tissue damage.

Both, animal histological studies (8, 41, 42) and human neuroimaging studies (37, 38), showed that it is possible to neuromodulate brain circuits without inducing tissue damage. The thermal index (TI) is the ratio of total acoustic power to the acoustic power required to raise tissue temperature by 1°C under defined assumptions. Finally, the non-thermal, mechanical bioeffect is described by the mechanical index (MI), which is directly proportional to the ultrasound beam's peak negative pressure and inversely proportional to the frequency of the beam.

The intensity, spatial-peak pulse-average (I_SPPA_) is the value of the pulse-average intensity at the point in the acoustic field where the pulse-average intensity is a maximum or is a local maximum within a specified region. The intensity, spatial-peak temporal-average (I_SPTA_) is the value of the temporal-average intensity at the point in the acoustic field where the temporal-average intensity is a maximum, or is a local maximum within a specified region.

The FDA guidelines defined the safety threshold for diagnostic usage of US for adult cephalic ultrasound, which can be applied to neuromodulation. These parameters are Isspa ≤ 190 W/cm^2^, Ispta ≤ 94 mW/cm^2^ and a mechanical index ≤ 1.9 (43).

### Focused Ultrasound for Targeted Drug Delivery

Focused ultrasound technique can be used also to facilitate drugs delivery in a specific brain area. Until now the most explored application is chemotherapy delivering. However, this versatile technique could be applied for neuromodulation purposes, with different mechanisms.

The first mechanism is a focal blood–brain barrier (BBB) opening, through a transient opening of endothelial tight junctions. Indeed, both animal (44, 45) and human (46) studies showed that FUS in combination with microbubbles administered intravenously can open the BBB, in a targeted, non-invasive, safe, and reversible manner. This technique could be used for targeted neuromodulation, with therapy which doesn't cross the BBB. For example Wang et al. (47) showed that it is possible to facilitate gene therapy delivery with recombinant adeno-associated virus, in a non-invasive way, through focused ultrasound targeted BBB opening, with potential applications for optogenetics (48) neuromodulation.

The second system is the local release of drugs, minimizing the effect on other brain areas. Indeed, focused ultrasound can be used to locally release drugs which are administered into the bloodstream through a vehicle (e.g., microbubble, liposome) sensitive to local temperature or pressure changes (49).

## Methods

The literature search methods included the PubMed/MEDLINE databases with the following research string, in Nov 2018: (“Neuromodulation” OR “Brain Stimulation”) AND (“focused ultrasound” OR HIFU OR LIFU OR Low-intensity focused ultrasound). After abstract reading and screening, only human studies which described focused ultrasound neuromodulation approaches were included in the present review. In addition to the search protocol described, further articles suggested by experts in the field where read and screened (Table 1).

## Results

### Physiological Effects in Normal Subjects

Legon et al. (10) used tFUS to target the human primary somatosensory cortex (S1), showing that tFUS significantly decreased the amplitudes of somatosensory evoked potentials elicited by median nerve stimulation. Furthermore, tFUS significantly modulated the spectral content of sensory-evoked brain oscillations and enhanced the performance on sensory discrimination tasks. The neurophysiologic effects had a spatial resolution of about 1 cm or less.

In another study, tFUS altered EEG intrinsic oscillatory dynamics, preferentially affecting the phase distribution of beta band and modulated the phase rate across beta and gamma frequencies. Furthermore, tFUS affected the phase distributions in the beta band of the early but not of the late components of somatosensory evoked potentials, suggesting a spatial specificity. This hypothesis was supported by the loss of neuromodulatory effects after the displacement of the transducer 1 cm laterally from the original cortical target (39).

Primary (SI) and secondary (SII) somatosensory cortical areas of the hand were targeted in a study by Lee et al. (50), in which two transducers were used. The areas were stimulated separately or simultaneously, under neuronavigation guide. tFUS elicited various types of tactile sensations in the contralateral hand/arm regions. The effects were transient and reversible, and the stimulation resulted safe, as assessed by repeated clinical and neuroradiological evaluations. In addition this study showed, the feasibility of the simultaneous stimulation of different human brain areas.

In Lee et al. (38), tFUS stimulation of the human somatosensory cortex elicited somatosensory sensations with anatomical specificity up to a finger, and evoked EEG potentials.

fMRI studies showed the effects of tFUS on cortical and subcortical brain areas, with the ability of high-resolution non-invasive functional brain mapping (33, 36, 37).

Indeed, Leo et al. (36), demonstrated that tFUS stimulation of cortical (primary motor cortex) and subcortical (head of the caudate) areas can induce blood oxygen level dependent (BOLD) signals in 3T and 7T fMRI, respectively. More recently, pairing tFUS on human primary motor cortex (M1) with 7T BOLD fMRI signals in a cued finger tapping task study, Ai et al. (33) showed that tFUS selectively increases BOLD activation volumes of the target finger representation. These effects did not spatially overcome the sonicated area, and therefore did not involve other motor regions, such as supplementary motor area (SMA) and dorsal premotor cortex (PMd).

tFUS has been used also to target the human primary visual cortex (V1) Lee et al. (37) showed, on BOLD fMRI signals, that tFUS stimulation elicited the activation of a network of brain regions, including V1 and other areas involved in visual and higher-order cognitive processes. Furthermore, stimulation elicited perception of phosphenes and EEG evoked responses.

The effects of tFUS on corticospinal excitability have also been studied through transcranial magnetic stimulation (TMS). Combining a custom-made FUS transducer and a 8-shaped TMS coil, Legon et al. (34) assessed for the first time in humans the effect of concentric and concurrent tFUS/TMS stimulation on M1. The stimulation had an inhibitory effect on single-pulse MEPs and intracortical facilitation, and significantly decreased the reaction time in a motor task.

Legon et al. (35) tested the effects tFUS stimulation on sensory thalamus, that was targeted by a single-element focused ultrasound through a neuronavigation system based on the individual subject anatomical MRI. tFUS stimulation inhibited the P14 SEP, and was associated with a change in EEG oscillatory dynamics and to a reduced ability in a tactile judgement task. In addition, this study outlined the value of taking into account the individual skull morphology to produce safe and accurate stimulations.

In a recent single-blind, sham-controlled study (40), tUS was targeted to the motor cortex through a diagnostic imaging ultrasound system. The unfocused stimulation increased MEPs amplitude by 34% compared to baseline, and the increase was recorded up to 6 min after the stimulation. This short-term increase of motor cortex excitability contrasts with a previous findings of MEP inhibition during concurrent tFUS/TMS (34). As discussed by the authors, stimulation parameters and other methodological factors might explain the different findings.

### Therapeutic Application

Despite several studies showed the neurological therapeutic applications of lesional FUS and FUS mediated BBB opening in different diseases like essential tremor (51–54), Parkinson's disease (55–57), depression (58, 59), obsessive-compulsive disorder (60, 61), neuropatic pain (62, 63), Alzheimer disease (46, 64), only two studies explored in humans the therapeutic efficacy of tUS (9) and tFUS (11) bioelectrical neuromodulation (Table 1).

Hameroff et al. (9) used a 8 MHz unfocused transducer to study the effects of transcranial ultrasound stimulation (tUS) on mood, and global affect in 31 patients with chronic pain, in a double-blind, sham-controlled crossover study. Stimulation was targeted to the posterior frontal cortex, contralateral to the most severe pain. After the stimulation, a significant improvement in subjective parameters of global affect derived from the Visual Analog Mood Scale was found.

As part of an ongoing clinical trial on low intensity focused ultrasound in acute brain injury (12), Monti et al. (11) reported a case of emergence from minimally conscious state after low intensity non-invasive ultrasonic thalamic stimulation.

### Transcranial Focused vs. Unfocused Ultrasound Neuromodulation

Despite transcranial focused ultrasound (tFUS) and transcranial unfocused ultrasound (tUS) neuromodulation techniques share the same basic mechanisms of action, when applied on the same target they can lead to quite different results.

These results are related to the intrinsic differences between the two techniques. The most important, one is the volume of the brain involved in the ultrasound field. It is intuitive that the volume of the brain involved in the focused or unfocused neuromodulation, and the underlying neural circuits, are crucial to determine the output of the tFUS or tUS neuromodulation. This has been supported also by experimental results, where tFUS and tUS were applied on the same target, the primary motor cortex: tUS increased MEPs amplitude (40) instead tFUS induced a MEP inhibition (34). In addition, the sonication delivered during the stimulus duration period, is generally continuous, without pauses, for tUS, and pulsed, characterized by pauses between the sonications, for tFUS. Low-intensity pulsed FUS is the most effective FUS technique for neuromodulation in both animal model (5, 6) and humans (Table 1). Instead, high intensity continuous FUS is widely used for therapeutic irreversible lesioning (51, 55, 58, 60).

## Discussion

Transcranial focused ultrasound is an emerging technique for non-invasive neurostimulation, with direct action on bioelettrical neural activity, and in addition could be used for targeted drug delivery.

Compared to magnetic or electric non-invasive brain stimulation, this technique has a higher spatial resolution and can reach deep structures. In addition, animal studies suggest that, potentially, different sites of the central and peripheral nervous system can be targeted by this technique.

Even if still in a small number, the increasing interest in this technique, led to encouraging results in human studies. These preliminary human studies focused their attention on classic non-invasive neurostimulation targets, like the primary motor cortex, somatosensory area or primary visual cortex, with some studies that explored deep structures like thalamus (11, 35) or basal ganglia (36). All showed neurostimulation efficacy in terms of clinical, neurophysiological or functional neuroradiological outcomes (Table 1).

The data collected since now shows that this technique is safe and well-tolerated, when the stimulation parameters and protocol follow the available guidelines. In addition, tFUS can be also conducted without hair shaving (65). The majority of the studies reported no severe adverse effects. Mild and moderate symptoms are reported such as neck pain, sleepiness, muscle twitches, itchiness, and headache (9, 34) (Table 1). In future studies, proper assessments, aimed to define the safety parameters for tUS and tFUS, are needed. Finally, every tUS or tFUS protocol should explore the role of auditory confounding factors on the neural responses, in order to show that the effect of stimulation is the consequence only of the targeted area neuromodulation, and not due to an indirect auditory impact (66, 67).

Overall, the results up to now encourage the study of tUS and tFUS as non-invasive neuromodulatory techniques in humans. The high spatial resolution of tFUS and the possibility of stimulating cortical and deep brain regions suggest many potential applications, such as cortical and subcortical mapping, the study of functional connectivity, the modulation of neurotransmission. Regarding tUS as a potential neuromodulatory tool, noteworthy is the high accessibility of the devices, which are routinely used in health care settings. Further research is needed to clarify tUS and tFUS efficacy and underlying mechanisms, and to optimize stimulation parameters and targeting accuracy. The initial safety profiles seem promising. A rigorous approach must be maintained in order to ensure safe sonications.

## Author Contributions

LB: conception, organization, execution, and writing of the first draft. EF: execution, writing of the first draft, and review and critique. VD: conception, organization, and review and critique.

### Conflict of Interest Statement

The authors declare that the research was conducted in the absence of any commercial or financial relationships that could be construed as a potential conflict of interest.
